# Insights Into the Regulation of Salt Tolerance by Flavonoid and Cyanoamino Acid Metabolism in Sorghum‐Sudangrass Hybrids

**DOI:** 10.1002/pld3.70186

**Published:** 2026-07-08

**Authors:** Yue Shi, Zili Liu, Shuancheng Liu, Wenjuan Chai, Junxiang Mu

**Affiliations:** ^1^ College of Life Science and Technology Jining Normal University Ulanqab China

**Keywords:** cyanoamino acid metabolism, flavonoid biosynthesis, salt tolerance, sorghum‐sudangrass hybrids, transcriptome

## Abstract

Clarifying the molecular mechanisms underlying salt tolerance in high‐performing sorghum‐sudangrass hybrids can aid the identification of salt tolerance genes. Here, the previously screened salt‐tolerant sorghum‐sudangrass hybrid variety MN9 and the salt‐sensitive variety MN4 were grown in sand‐filled pots. At the five‐leaf stage (21 days after sowing), seedlings were subjected to NaCl solutions at six concentrations (0, 70, 140, 210, 280, and 350 mM). After 72 h of treatment, mature leaves were collected to determine physiological indices across all concentrations. We then conducted transcriptome sequencing of leaf samples from the 0 mM (control) and 280 mM (high salt stress) treatments, and the sequencing results were validated by qRT‐PCR. Under salt stress, MN9 maintained a high relative chlorophyll content (SPAD value), accumulated significantly more proline than MN4, and showed high peroxidase (POD) activity throughout the treatment period. Transcriptome profiling revealed 4340 differentially expressed genes (DEGs) between the salt‐tolerant and salt‐sensitive varieties under salt stress, including 2624 upregulated and 1716 downregulated genes. KEGG enrichment analysis showed that, under control conditions, genes in both varieties were enriched in the photosynthesis pathway; SORBI_3001G147400 (*petF*) supported normal photosynthetic function. Following salt stress, DEGs were predominantly enriched in biosynthesis of secondary metabolites, cyanoamino acid metabolism, and flavonoid biosynthesis. Key flavonoid biosynthesis genes (e.g., *CHS*, *CYP98A*, and *CYP75B1*) were specifically induced. In the cyanoamino acid metabolism pathway, SORBI_3004G335500 (*HNL*) and SORBI_3001G012400 (*UGT85B1*) were strongly expressed in MN9. The hydroxynitrile lyase gene *HNL* (SORBI_3004G335500) was experimentally verified as a key regulator of salt tolerance in sorghum‐sudangrass hybrid.

## 1 Introduction

Soil salinization is a global challenge that threatens agricultural production and ecological sustainability, as it is a major abiotic stress that limits crop growth (Parida and Das 2005). Saline–alkali soils cover more than 6% of the world's total land area (Huang et al. 2025); soil salinization hinders sustainable agriculture, severely reduces crop yields, and decreases productivity (Bhat et al. 2020; Charles et al. 2019). Numerous studies have shown that salt stress harms plants through multiple pathways, including reduced photosynthetic capacity, water balance disruption, alterations in ion concentrations, inhibition of plasma membrane antioxidation, and interference with the synthesis of osmotic adjustment substances (Gulzar et al. 2003; Parida et al. 2004; Rubio et al. 2020).

RNA sequencing (RNA‐seq) technology has been widely used to investigate the responses of plants to salt stress, including identifying differentially expressed genes (DEGs) in forage grasses (Chai et al. 2025), characterizing stress‐responsive gene families in crops (Qu et al. 2025; Zhuang et al. 2025), mining key salt tolerance genes (Wang et al. 2023), and elucidating the molecular mechanisms underlying salt stress responses (Zhang et al. 2025). Previous studies indicate that upregulation of genes related to flavonoid and proline biosynthesis can enhance salt tolerance in *Arabidopsis thaliana* (Wang et al. 2016), and increased expression of flavonoid synthase genes can further enhance salt tolerance in *A. thaliana* (Zhang et al. 2023). In carrots, flavonoid pathway genes (3′‐hydroxylase and chalcone isomerase [CHI]) were first identified as key regulatory factors mediating salt tolerance (Klimek‐Chodacka et al. 2016). Cyanoalanine synthase (CAS), a key enzyme in plant cyanide detoxification, can also significantly accelerate cyanide metabolism and enhance salt tolerance in *A. thaliana* (Xu et al. 2023).

Sorghum‐sudangrass hybrids ( *Sorghum bicolor* × *S. sudanense* ) are interspecific hybrids derived from *S. bicolor* (Linn.) Moench and *S. sudanense* (Piper) Stapf., both in the family Poaceae. As a high‐quality, high‐yielding forage grass, it combines strong adaptability, high productivity, and marked salt–alkali tolerance, while also contributing to ecological protection and supporting the development of animal husbandry (McCormick et al. 2018; Nawaz et al. 2010; Pupo et al. 2022). Studies of sorghum have shown that high salinity inhibits seed germination and suppresses seedling growth and development (Almodares et al. 2007; Chauhan et al. 2012). Quantitative trait loci associated with salt tolerance during germination and seedling stages have been identified using sorghum recombinant inbred lines (Wang et al. 2014). An ultrahigh‐density genetic linkage map has been constructed for sorghum‐sudangrass hybrids (Lu et al. 2022), and significant variation in salt tolerance levels has been documented among different sorghum‐sudangrass hybrid varieties (Nawaz et al. 2010; Maiti et al. 1994).

Despite these advances, transcriptome‐level studies of salt tolerance in sorghum‐sudangrass hybrids remain limited compared with model forage species such as alfalfa. As a result, the core molecular pathways and key regulatory genes controlling its salt stress response are still poorly understood, which limits our understanding of underlying mechanisms and molecular breeding applications. In this study, prescreened salt‐tolerant and salt‐sensitive sorghum‐sudangrass hybrid varieties were used to determine the expression of salt tolerance genes and investigate the underlying molecular mechanisms via transcriptome sequencing under salt stress. Our goal was to evaluate the salt tolerance potential of sorghum‐sudangrass hybrids and provide a technical reference for breeding salt‐tolerant varieties and improving the efficient utilization of saline–alkali lands.

## 2 Materials and Methods

### 2.1 Plant Materials and Growth Conditions

Based on the preliminary screening of a large collection of sorghum‐sudangrass ( *S. bicolor* × *S. sudanense* ) inbred lines, the salt‐tolerant variety MN9 and the salt‐sensitive variety MN4 were selected as experimental materials. The experiment was conducted in 2024 in an artificial climate chamber at the Botanical Garden of Jining Normal University under controlled conditions of 26°C with a 12‐h light/12‐h dark photoperiod. A pot culture method was employed using plastic pots (20.5‐cm diameter × 16.5‐cm height), each filled with 0.7 kg of sieved fine sand. Soil moisture was determined gravimetrically. Seeds were sown when the sand water content reached 20%, with 10 seeds per pot. After emergence, seedlings were thinned to two per pot.

When seedlings reached the five‐leaf stage (approximately 21 days after emergence), 500 mL of salt solution was slowly applied to each pot to ensure uniform permeation throughout the sand layer. Both MN9 (salt‐tolerant) and MN4 (salt‐sensitive) varieties were subjected to NaCl treatments at concentrations of 0 (distilled water control), 70, 140, 210, 280, and 350 mM. Distilled water was supplemented daily based on gravimetric measurements to maintain the sand water content at 20% and prevent dilution of salt concentrations. Leaf samples were collected 72 h after treatment, with three biological replicates per treatment (each replicate comprised 10 seedlings with uniform growth).

### 2.2 Determination of Physiological Indicators

For each treatment, 20 mature leaves of similar size exhibiting vigorous growth were selected from the middle portion of seedlings in both control and stress groups. Relative chlorophyll content (SPAD value) was measured using a portable chlorophyll meter (SPAD‐502, Minolta). Peroxidase (POD) activity and the proline (Pro) content were determined using commercial assay kits (Nanjing Jiancheng Bioengineering Institute, Nanjing, China). The flavonoid content was quantified by visible spectrophotometry following the manufacturer's protocol (Solarbio, Beijing, China).

### 2.3 RNA Extraction and Transcriptome Sequencing

Total RNA was extracted from leaf tissue using a plant RNA extraction kit, and genomic DNA contamination was removed by DNase I digestion. RNA quality was initially assessed by 1.0% agarose gel electrophoresis, with band integrity visualized using a UV gel imaging system. Samples meeting the following quality criteria were selected for downstream analysis: OD_260_/OD_280_ ≥ 1.8 and OD_260_/OD_230_ ≥ 2.0. Messenger RNA was enriched using oligo (dT) magnetic beads and subsequently fragmented to approximately 300 bp. First‐ and second‐strand cDNA synthesis was performed by reverse transcription, and sample‐specific index sequences were ligated to each library. Target‐size fragments were recovered by agarose gel electrophoresis and amplified by PCR to complete library construction. High‐throughput sequencing was performed on the Illumina HiSeq 2500 platform.

Raw sequencing data were subjected to filtering processing using Trimmomatic software (Bolger et al. 2014). Low‐quality reads and adapter sequences were eliminated through the evaluation of base quality, GC content distribution, and sequencing error rate, thus yielding high‐quality clean reads. To guarantee the reliability of sequencing data, FastQC software was employed to conduct quality control assessment on indicators including GC content and Q30 of the clean reads. Subsequently, the clean reads were mapped to the sorghum reference genome *Sorghum_bicolor_*v3.1genome.fa (GenBank accession No.: GCA_000003195.3) with HISAT2 v2.1.0 software (Pertea et al. 2016), and the genome mapping information of reads was obtained and generated into SAM format files. Novel transcript assembly was implemented by StringTie software, and gene functional annotation and metabolic pathway enrichment analysis were further performed on the basis of GO, KEGG, and other public databases.

### 2.4 Screening and Validation of DEGs

#### 2.4.1 Quantification of Gene Expression

Gene expression levels were quantified using FPKM (Fragments Per Kilobase of exon model per Million mapped reads). The FPKM value is calculated as follows:

$$\text{FPKM}=\frac{\text{total exon fragments}}{\text{Mapped reads}\;\left(\text{Millions}\right)\times \text{exon length}\left(\text{KB}\right)}$$

#### 2.4.2 Transcriptome Data Analysis

DEGs were identified through pairwise comparisons using the R package DESeq2 (Love et al. 2014), with screening thresholds of |log_2_FC| ≥ 1 and FDR ≤ 0.05. Fold change (FC) represents the ratio of expression levels between two samples or groups. The false discovery rate (FDR) was derived by adjusting *p*‐values for multiple comparisons and served as the primary criterion for DEG identification. KEGG pathway enrichment analysis was performed using KOBAS 3.0, and heatmaps were generated with TBtools.

#### 2.4.3 Quantitative Real‐Time PCR (qRT‐PCR)

To validate the transcriptome sequencing results, four DEGs were randomly selected for qRT‐PCR analysis. Primer sequences are listed in Table 1. Primers were designed using Primer 6.0, with amplicon lengths ranging from 80 to 300 bp. The thermal cycling conditions were as follows: initial denaturation at 95°C for 5–10 min, followed by 35–40 cycles of denaturation at 95°C, annealing at 58°C–62°C for 15–30 s, and extension at 72°C for 20–30 s. Each reaction was performed in triplicate to ensure reproducibility.

**TABLE 1:** RT‐qPCR primers for salt tolerance‐related genes in *Sorghum bicolor* × *S. sudanense* .

Gene name	GenBank number	Forward primer
LOC8081476	SORBI_3001G417200	F: TTCGACTTCAACCTGACGCT R: GGGCGTGAAGTAGAGCTTGT
LOC8082405	SORBI_3003G327800	F: CGCTCTTCACTCTCAGGGAC R: GAGATCACCGTCGTGTCAGT
LOC8056975	SORBI_3005G136200	F: TGAGATGAAGTACCACGCCG R: GATCGACGACTGGTGGTTGT
LOC110435085	SORBI_3004G335500	F: CCACAGCTCTCCCAAGTTGT R: TCTTCGTGGTCGTTGGTCAG
LOC8076587	SORBI_3007G043200	F: CCACAGCTCTCCCAAGTTGT R: TCCGGCAAATTGTCATCCCA
LOC8064486	SORBI_3006G146000	F: TACGAGCCGCTGACGAATAC R: GCATCGCTCTCGGGTAATCA
18S rRNA gene	XM_002468234.1	F: CATTCGCCAAGGATGTTTTC R: TTGTCTGGACCTGGTGAGTTT

### 2.5 Statistical Analysis

Raw data were organized using Microsoft Excel 2021. Physiological parameters, including relative chlorophyll content, POD activity, and proline content, were analyzed by two‐way analysis of variance (two‐way ANOVA) using SPSS 19.0. Post hoc multiple comparisons were performed using Duncan's multiple range test, with statistical significance set at *p* < 0.05. Figures were generated using GraphPad Prism 10.1.2, and significant differences among groups were denoted by lowercase letters (a, b, and c).

## 3 Results and Analysis

### 3.1 Effects of Salt Stress on the Physiological Indexes and Phenotypic Traits of Sorghum‐Sudangrass

Six NaCl concentration gradients (0, 70, 140, 210, 280, and 350 mM) were established in this experiment. POD activity in MN9 increased progressively as the salt concentration increased, whereas MN4 exhibited a fluctuating pattern of increases and decreases. At 280‐mM NaCl, POD activity in MN4 increased to 714.29 U/g, approaching that of MN9 (707.55 U/g) with no significant difference between the two varieties. Following exposure to 350‐mM NaCl, POD activity in MN9 reached a peak of 762.46 U/g, whereas that of MN4 declined and was significantly lower than that of MN9. These results suggest that the antioxidant system of MN9 was continuously activated under moderate to high salt stress (280–350 mM), conferring enhanced stress adaptability. In contrast, the antioxidant response in MN4 was delayed and unstable, which was insufficient to maintain long‐term salt tolerance.

**FIGURE 1:**
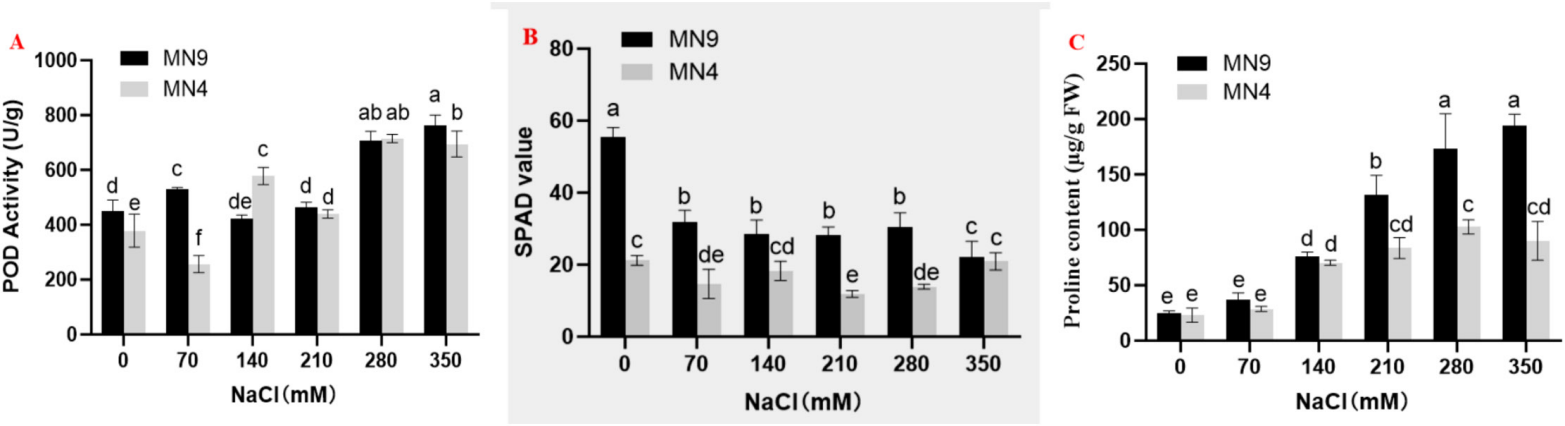
Relative POD activity, SPAD value, and proline content of sorghum‐sudangrass under salt stress. *Note:* Error bars represent standard deviation (SD). Statistical significance was determined by one‐way analysis of variance (ANOVA) followed by Duncan's multiple range test; different lowercase letters (a, b, and c) indicate significant differences (*p* < 0.05).

The relative chlorophyll content in sorghum‐sudangrass leaves exhibited a gradual declining trend overall (Figure 1). The relative chlorophyll content of the salt‐tolerant cultivar MN9 was significantly higher than that of the salt‐sensitive cultivar MN4 across treatments. After 280‐mM NaCl treatment, the relative chlorophyll content in MN9 decreased by 44.95% compared with the control, whereas MN4 showed a 34.44% reduction. The free proline content in both cultivars increased as the salt concentration increased. No significant differences were observed between the two cultivars at 0‐, 70‐, and 140‐mM NaCl; however, the proline content was significantly higher in MN9 than in MN4 under moderate to high salt stress (210, 280, and 350 mM). These results indicate that the salt‐tolerant cultivar accumulated more proline under salt stress, suggesting that the salt‐tolerant cultivar had a greater osmoregulatory capacity compared with the salt‐sensitive cultivar.

In conclusion, 280‐mM NaCl represents a critical threshold for distinguishing salt tolerance characteristics between the two cultivars. At this concentration, although MN4 was capable of short‐term antioxidant system activation, its capacity to maintain photosynthetic pigments and accumulate osmoprotectants was inferior to that of MN9.

**FIGURE 2:**
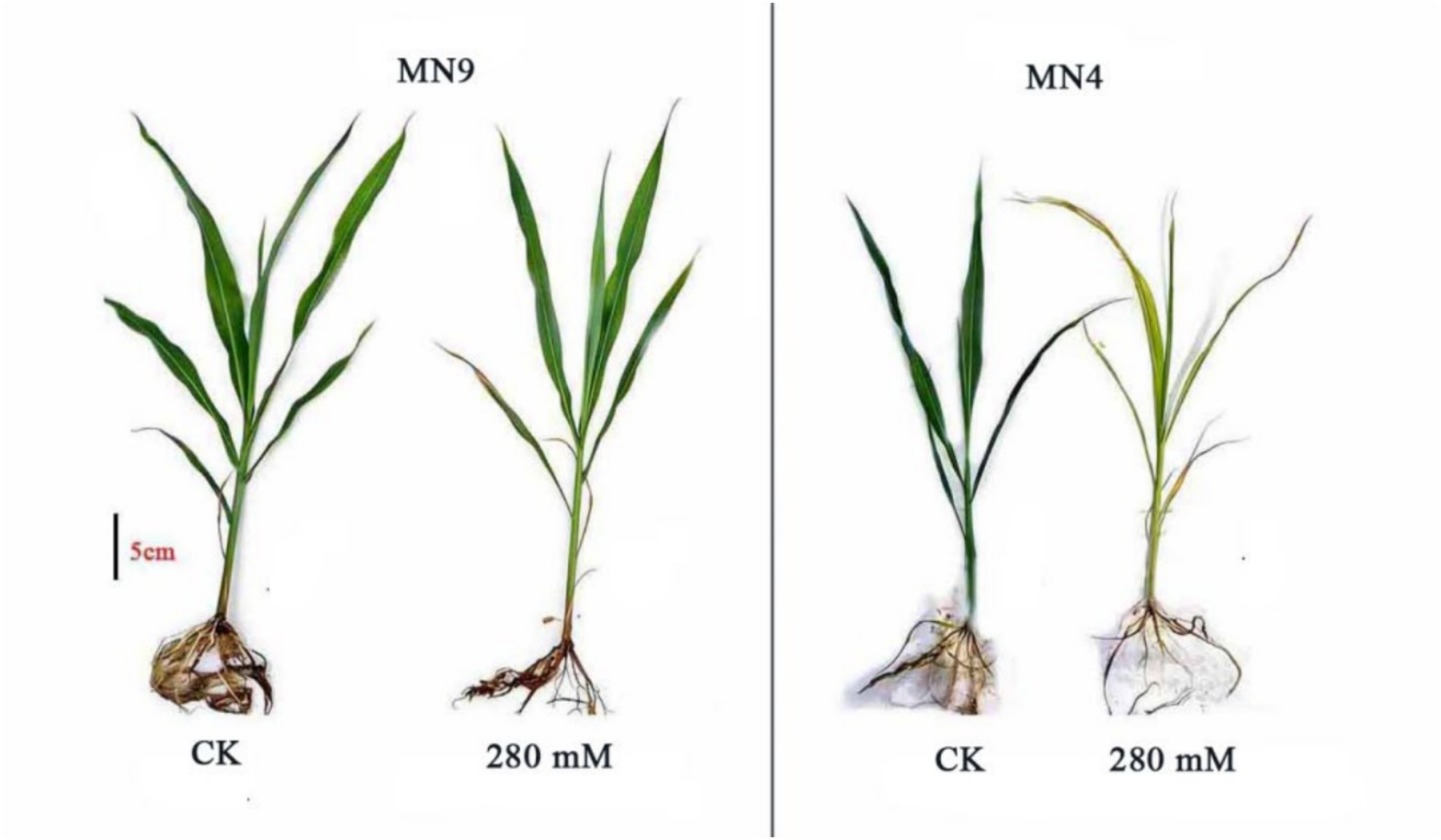
Phenotypic characteristics of two sorghum‐sudangrass cultivars under salt stress. *Note:* The plants of MN9 and MN4 lines are shown under the control (CK, 0‐mM NaCl) and salt stress (280‐mM NaCl) treatments. The scale bar represents 5 cm.

Based on the physiological results described above, two salt stress levels (0‐ and 280‐mM NaCl) were selected for phenotypic characterization of sorghum‐sudangrass (Figure 2). Under control conditions, MN9 plants were taller with darker green leaves and greater numbers of leaves, exhibiting overall superior growth compared with MN4. Following salt stress treatment (280‐mM NaCl), both cultivars showed significantly reduced growth relative to their respective controls, with marked inhibition of both shoot and root development. MN9‐0‐L displayed bright green, fully expanded leaves with a large surface area. MN9‐280‐L exhibited slight chlorosis with mild yellow‐brown spotting along leaf margins, indicative of mild salt injury. In contrast, MN4‐0‐L had dark green leaves with a narrower shape and fewer leaves overall. MN4‐280‐L displayed pronounced yellowing, leaf desiccation, wilting, and curling, characteristic of severe salt damage.

These observations suggest that sorghum‐sudangrass adapts to high salt stress in part by reducing leaf area, thereby decreasing transpirational water loss and nutrient demand. Furthermore, the greater degree of chlorosis observed in MN4‐280‐L compared with MN9‐280‐L indicates that the salt‐tolerant cultivar MN9 possesses superior salt tolerance mechanisms. MN9 more effectively maintained normal leaf physiological function under salt stress, mitigating chlorosis and leaf area reduction, and thus demonstrated stronger overall adaptability to saline conditions.

### 3.2 Transcriptome Sequencing Data Assembly and Annotation

Quality assessment results of transcriptome data from leaves of the two contrasting salt‐tolerant sorghum‐sudangrass cultivars are presented in Table 2. After quality filtering, a total of 33,819,304–60,538,792 clean reads were obtained across all samples. Alignment to the reference genome yielded mapping rates ranging from 68.21% to 97.71%. The GC content ranged from 48.22% to 52.44%, Q20 values exceeded 98.76%, and Q30 values were above 95.56% for all samples. These metrics indicate that the sequencing data were of high quality and sufficient reliability for subsequent bioinformatics analyses.

**TABLE 2:** Summary of RNA‐seq data quality for each sample.

Sample name	Clean reads	Mapped reads	Mapped_Rate (%)	GC%	Q20%	Q30%
MN4‐0‐L‐1	39,281,694	38,382,143	97.71	51.16	99.11	97.42
MN4‐0‐L‐2	33,819,304	32,970,439	97.49	52.44	99.17	97.58
MN4‐0‐L‐3	38,178,312	37,273,486	97.63	50.46	99.16	97.63
MN4‐280‐L‐1	60,538,792	57,929,570	95.69	49.01	99.22	97.78
MN4‐280‐L‐2	38,877,366	36,754,661	94.54	49.27	99.21	97.76
MN4‐280‐L‐3	48,409,486	46,197,172	95.43	48.22	99.22	97.84
MN9‐0‐L‐1	47,336,898	33,694,403	71.18	51.58	99.09	97.49
MN9‐0‐L‐2	48,463,424	44,373,111	91.56	52.02	99.07	97.44
MN9‐0‐L‐3	41,624,480	38,078,074	91.48	51.76	99.05	97.37
MN9‐280‐L‐1	53,746,696	48,678,382	90.57	51.79	99.14	97.56
MN9‐280‐L‐2	39,912,872	36,125,140	90.51	51.96	98.83	96.92
MN9‐280‐L‐3	48,072,650	43,592,279	90.68	49.91	99.19	97.76

### 3.3 Screening of DEGs in Sorghum‐Sudangrass Leaves

**FIGURE 3:**
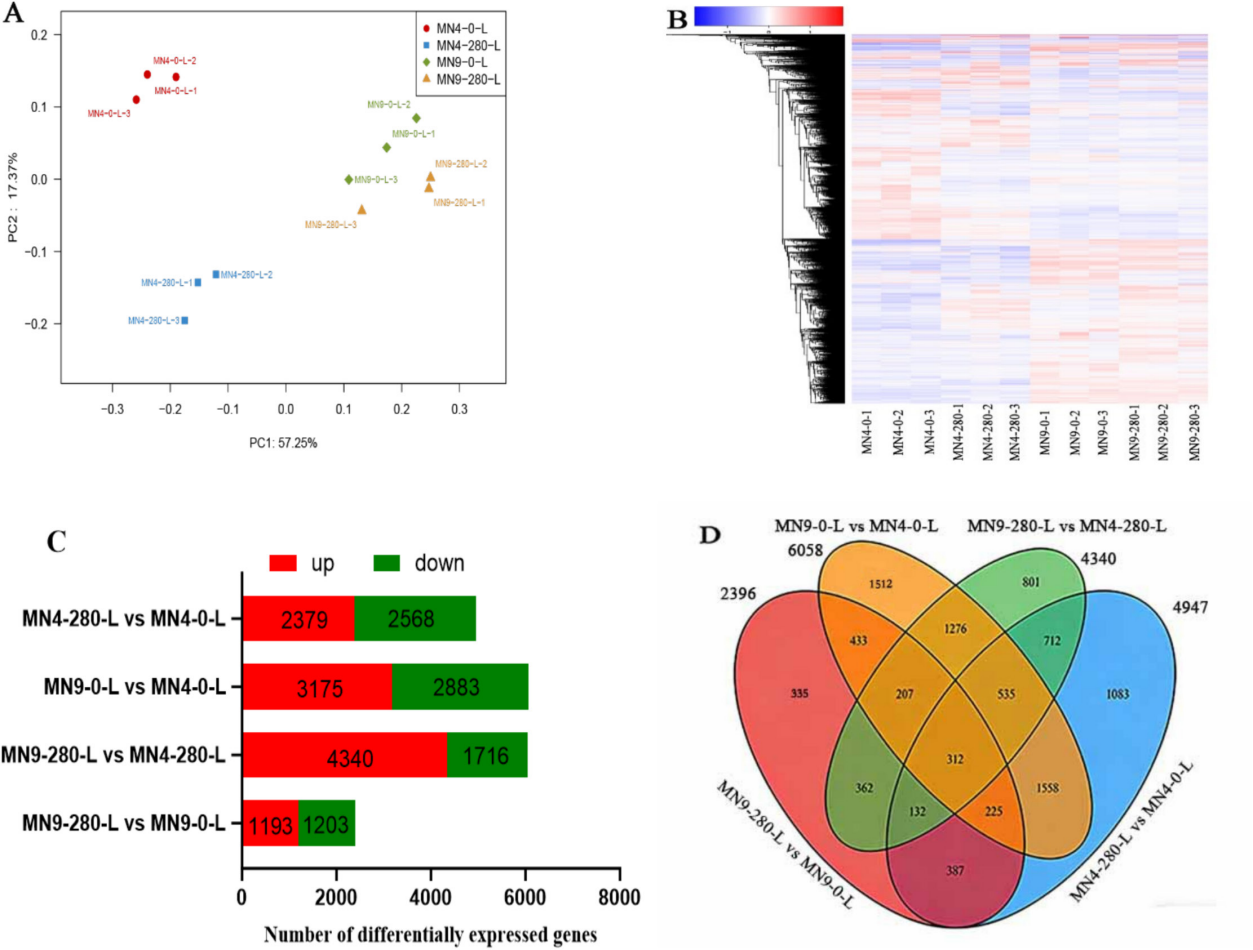
(A) Principal coordinate analysis (PCoA) based on Euclidean distances of gene expression profiles. (B) Hierarchical clustering analysis of differentially expressed genes (DEGs). (C) Number of DEGs identified in leaves after 72 h of 280‐mM salt treatment. Red indicates upregulated genes, green indicates downregulated genes, and the numbers indicate gene counts. (D) Venn diagram showing DEGs in leaves of different sorghum‐sudangrass cultivars under 280‐mM salt treatment. Overlapping regions represent shared genes between cultivars.

To investigate the molecular mechanisms underlying salt tolerance in sorghum‐sudangrass, RNA‐Seq was employed to characterize the transcriptomes of MN4 and MN9 under control and salt stress conditions. Principal coordinate analysis (PCoA) based on Euclidean distances of gene expression profiles revealed significant separation between treatments for both cultivars, with strong reproducibility within each treatment group. Significant separation was also observed between the leaves of salt‐tolerant and salt‐sensitive cultivars under salt stress. The first principal coordinate (PCo1) explained 57.25% of the total variance, indicating that salt treatment was the primary factor driving transcriptomic differences (Figure 3A). Hierarchical clustering analysis was performed based on Pearson correlation coefficients calculated from gene expression levels (FPKM values) across all samples (Figure 3B). Clear separation was observed between the control and salt‐treated groups. Additionally, the three biological replicates for each treatment clustered tightly together, confirming high sample reproducibility and reliable quantification of gene expression differences.

Differential expression analysis revealed distinct transcriptomic responses between the two cultivars following salt stress. After 280‐mM NaCl treatment, 2396 DEGs were identified in leaves of salt‐tolerant MN9, comprising 1193 upregulated DEGs and 1203 downregulated DEGs. In contrast, 4947 DEGs were identified in salt‐sensitive MN4, including 2379 upregulated and 2568 downregulated genes. These results indicate that salt‐sensitive MN4 exhibited a substantially greater transcriptomic response to salt stress than salt‐tolerant MN9, with slightly more genes downregulated than upregulated. Between these two cultivars, 6058 DEGs (3175 upregulated and 2883 downregulated) were identified under control conditions, compared with 4340 DEGs (2624 upregulated and 1716 downregulated) under salt stress, indicating that intercultivar transcriptomic differences were reduced following salt treatment (Figure 3C).

To identify shared and cultivar‐specific genes associated with the salt stress response, a Venn diagram analysis was performed (Figure 3D). Following 280‐mM NaCl treatment, 1083 cultivar‐specific DEGs were detected in MN4 leaves compared with only 335 in MN9 leaves. The substantially higher number of salt stress‐responsive DEGs unique to salt‐sensitive MN4 suggests that this cultivar may rely on a broader, less specific transcriptional response to cope with stress, whereas MN9 may achieve efficient salt tolerance through a smaller set of key regulatory genes. Under control conditions, 1512 cultivar‐specific DEGs were identified between the two cultivars, compared with 801 under salt stress. A total of 312 DEGs were shared between cultivars across both control and salt stress conditions, representing 1.78% of all identified DEGs.

### 3.4 GO and KEGG Enrichment Analyses of DEGs Between Two Cultivars Under Salt Stress

#### 3.4.1 GO Enrichment Analysis of DEGs

Gene Ontology (GO) enrichment analysis was performed on DEGs from the two sorghum‐sudangrass genotypes under salt stress. The top 10 most significantly enriched terms in each comparison revealed that all three major GO categories, cellular component (CC), biological process (BP), and molecular function (MF), were substantially affected by salt stress (Table 3). Comparison between cultivars (MN9‐280‐L vs. MN4‐280‐L and MN9‐0‐L vs. MN4‐0‐L) revealed that under salt stress, MN9 was significantly enriched in terms related to response to water deprivation, response to oxygen‐containing compounds, and processes associated with osmotic adjustment and oxidative stress. In contrast, MN4 showed greater enrichment in GO terms related to chloroplast structure. Notably, even under control conditions, MN9 exhibited enrichment in BPs such as response to abiotic stimulus and stress response, with genes associated with oxidoreductase activity significantly upregulated. These findings suggest that MN9 possesses inherently stronger stress perception and antioxidant capacity, adapting to salt stress through metabolic and regulatory mechanisms, whereas MN4 primarily exhibits a passive response pattern characterized by structural damage and repair.

**TABLE 3:** GO enrichment of DEGs under salt stress in sorghum‐sudangrass hybrids.

Comparison group	GO ID	Core term	Category	*p*	FDR	Gene ratio	Upregulated	Downregulated
MN9‐280‐L vs. MN4‐280‐L	GO:0009266	Response to temperature stimulus	BP	5.29E‐06	0.008275	102/1331	72	30
	GO:0009414	Response to water deprivation	BP	9.10E‐06	0.007117	65/1331	52	13
	GO:0009415	Response to water	BP	9.85E‐06	0.005136	66/1331	52	14
	GO:1901700	Response to oxygen‐containing compound	BP	1.33E‐05	0.005186	214/1331	151	63
	GO:0001101	Response to acid chemical	BP	2.92E‐05	0.009147	168/1331	123	45
	GO:0006950	Response to stress	BP	4.63E‐05	0.012079	346/1331	230	116
	GO:0010035	Response to inorganic substance	BP	0.000121	0.027039	131/1331	90	41
	GO:0050896	Response to stimulus	BP	0.000124	0.024247	561/1331	360	201
	GO:0010109	Regulation of photosynthesis	BP	0.000205	0.035645	14/1331	6	8
	GO:0009409	Response to cold	BP	0.000263	0.041143	69/1331	49	20
MN9‐0‐L vs. MN4‐0‐L	GO:0009628	Response to abiotic stimulus	BP	7.41E‐06	0.015707	394/2023	245	149
	GO:0031226	Intrinsic component of plasma membrane	CC	2.95E‐05	0.031218	94/2023	36	58
	GO:0009266	Response to temperature stimulus	BP	3.75E‐05	0.026459	139/2023	88	51
	GO:0009409	Response to cold	BP	7.74E‐05	0.040984	101/2023	61	40
	GO:0006950	Response to stress	BP	0.000129	0.054808	504/2023	281	223
	GO:0031224	Intrinsic component of membrane	CC	0.000211	0.074566	161/2023	62	99
	GO:0009611	Response to wounding	BP	0.000434	0.131485	70/2023	40	30
	GO:0016491	Oxidoreductase activity	MF	0.000551	0.145956	174/2023	111	63
	GO:0005887	Integral component of plasma membrane	CC	0.000823	0.193821	60/2023	18	42
MN9‐280‐L vs. MN9‐0‐L	GO:0003700	DNA‐binding transcription factor activity	MF	4.20E‐05	0.043153	93/714	35	58
	GO:0009415	Response to water	BP	0.000248	0.127292	37/714	15	22
	GO:1901700	Response to oxygen‐containing compound	BP	0.000259	0.088639	118/714	48	70
	GO:0140110	Transcription regulator activity	MF	0.000525	0.13491	94/714	35	59
	GO:0051119	Sugar transmembrane transporter activity	MF	0.000577	0.118702	12/714	4	8
	GO:0009605	Response to external stimulus	BP	0.000699	0.119785	107/714	42	65
	GO:0009733	Response to auxin	BP	0.001085	0.15936	34/714	14	20
	GO:0031226	Intrinsic component of plasma membrane	CC	0.00131	0.168316	36/714	11	25
	GO:0004553	Hydrolase activity, hydrolyzing O‐glycosyl compounds	MF	0.001413	0.16141	27/714	6	21
	GO:0006355	Regulation of transcription, DNA‐templated	BP	0.001484	0.152605	117/714	50	67
MN4‐280‐L vs. MN4‐0‐L	GO:0009532	Plastid stroma	CC	9.35E‐07	0.001576	128/1626	117	11
	GO:0009570	Chloroplast stroma	CC	1.85E‐06	0.00156	125/1626	114	11
	GO:0009507	Chloroplast	CC	1.14E‐05	0.006383	311/1626	255	56
	GO:0009536	Plastid	CC	1.23E‐05	0.005187	321/1626	263	58
	GO:0009658	Chloroplast organization	BP	0.000148	0.04998	44/1626	43	1
	GO:0009628	Response to abiotic stimulus	BP	0.000158	0.044451	311/1626	172	139
	GO:0031226	Intrinsic component of plasma membrane	CC	0.000163	0.039305	75/1626	17	58
	GO:0009657	Plastid organization	BP	0.000219	0.046119	54/1626	50	4
	GO:0004553	Hydrolase activity, hydrolyzing O‐glycosyl compounds	MF	0.000393	0.073627	54/1626	16	38
	GO:0009266	Response to temperature stimulus	BP	0.000413	0.069697	109/1626	64	45

Within‐cultivar comparisons (MN4‐280‐L vs. MN4‐0‐L and MN9‐280‐L vs. MN9‐0‐L) revealed that DEGs in MN4 under salt stress were significantly enriched in CC terms, including plastid stroma and chloroplast organization. In contrast, DEGs in MN9 were enriched in MF terms such as DNA‐binding transcription factor activity and transcription regulator activity, as well as BP terms including response to water and response to oxygen‐containing compounds. These results indicate that MN9 achieves precise transcriptional reprogramming and osmotic adjustment primarily through the regulation of transcription factors and metabolic enzymes under salt stress, whereas MN4 appears to undergo substantial photosynthetic apparatus damage, activating numerous genes to counteract stress‐induced chloroplast dysfunction.

#### 3.4.2 KEGG Enrichment Analysis of DEGs

**FIGURE 4:**
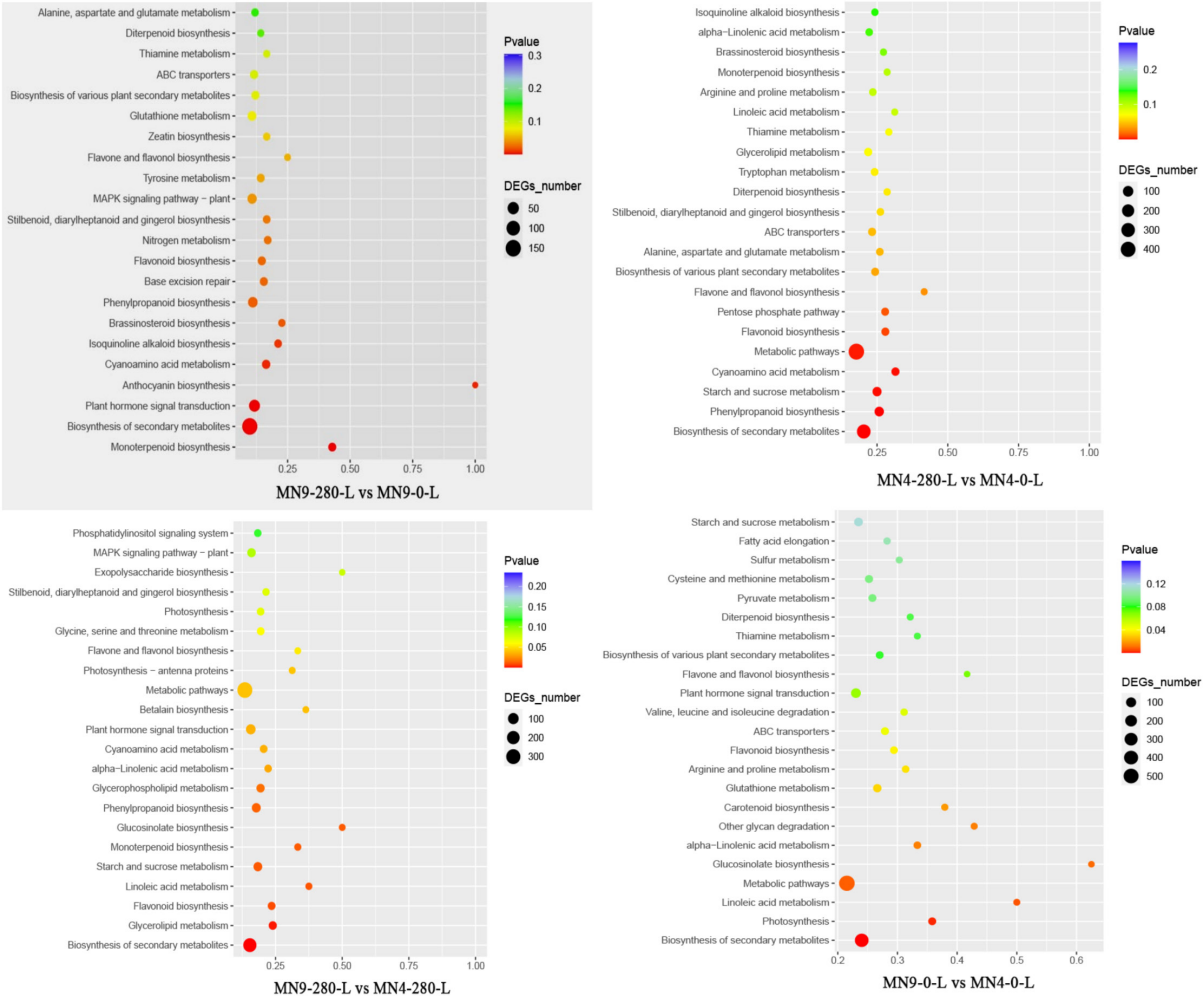
KEGG pathway enrichment analysis results of differentially expressed genes (DEGs) in sorghum‐sudangrass.

KEGG pathway enrichment analysis revealed that DEGs identified in within‐cultivar comparisons (MN9‐280‐L vs. MN9‐0‐L and MN4‐280‐L vs. MN4‐0‐L) were predominantly enriched in biosynthesis of secondary metabolites, cyanoamino acid metabolism, and flavonoid biosynthesis. Between‐cultivar comparisons (MN9‐280‐L vs. MN4‐280‐L and MN9‐0‐L vs. MN4‐0‐L) identified shared enrichment in biosynthesis of secondary metabolites, linoleic acid metabolism, and flavonoid biosynthesis, whereas glycerolipid metabolism and photosynthesis were distinctly enriched pathways (Figure 4). Both cultivars showed enrichment in photosynthesis‐related pathways under control conditions, suggesting that differences in salt tolerance between the two cultivars may be associated with photosynthetic capacity. Under salt stress, both cultivars were enriched in biosynthesis of secondary metabolites and flavonoid biosynthesis pathways. Notably, cyanoamino acid metabolism was enriched in both cultivars only following salt treatment and was absent in control groups, indicating that this pathway may play a role in the salt tolerance mechanism of sorghum‐sudangrass.

### 3.5 Photosynthesis

**FIGURE 5:**
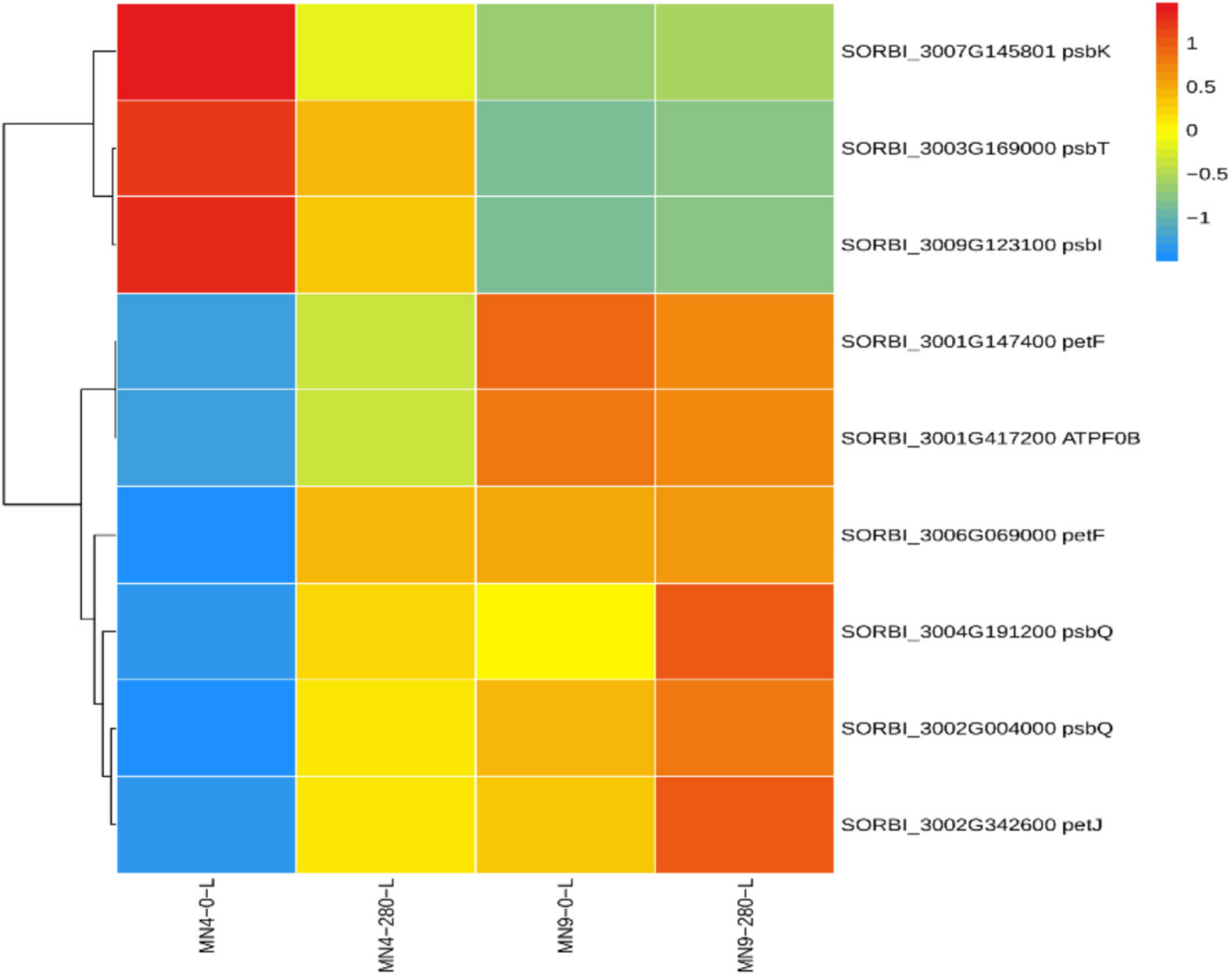
Heatmap of expression patterns for photosynthesis‐related salt tolerance candidate genes. *Note:* The heatmap displays row‐wise Z‐score normalized values based on log_2_(FPKM) values. Red indicates expression levels above the average level for the gene, whereas blue indicates expression levels lower than the average level.

Photosynthesis is fundamental to crop growth and yield, and its inhibition under salt stress is a primary contributor to yield reduction. To identify key photosynthesis‐related genes associated with salt tolerance in sorghum‐sudangrass, nine candidate genes were screened and analyzed. These genes encode proteins involved in multiple photosynthetic processes, including PSII reaction center proteins, oxygen‐evolving complex proteins, cytochrome b6f complex components, ferredoxin, and ATP synthase subunits (Figure 5), spanning light energy capture, electron transport, and energy conversion. SORBI_3001G147400 (*petF*) exhibited a characteristic salt tolerance‐associated expression pattern, with significantly higher expression levels in salt‐tolerant MN9 compared with salt‐sensitive MN4 under salt stress. Ferredoxin plays a critical role in photosynthetic electron transport, and its specific induction in the salt‐tolerant cultivar likely contributes to maintaining electron transport chain stability under saline conditions, thereby exerting a positive regulatory effect. Additionally, SORBI_3003G169000 and SORBI_3009G123100 were significantly downregulated in salt‐sensitive MN4 under salt stress but remained stably expressed in salt‐tolerant MN9, potentially facilitating adaptation to saline conditions. The distinct expression patterns of these genes between cultivars make them promising candidate genes for elucidating the molecular mechanisms underlying salt tolerance in sorghum‐sudangrass.

### 3.6 The Flavonoid Biosynthesis Pathway Is Specifically Activated in the Salt‐Tolerant Cultivar MN9

**FIGURE 6:**
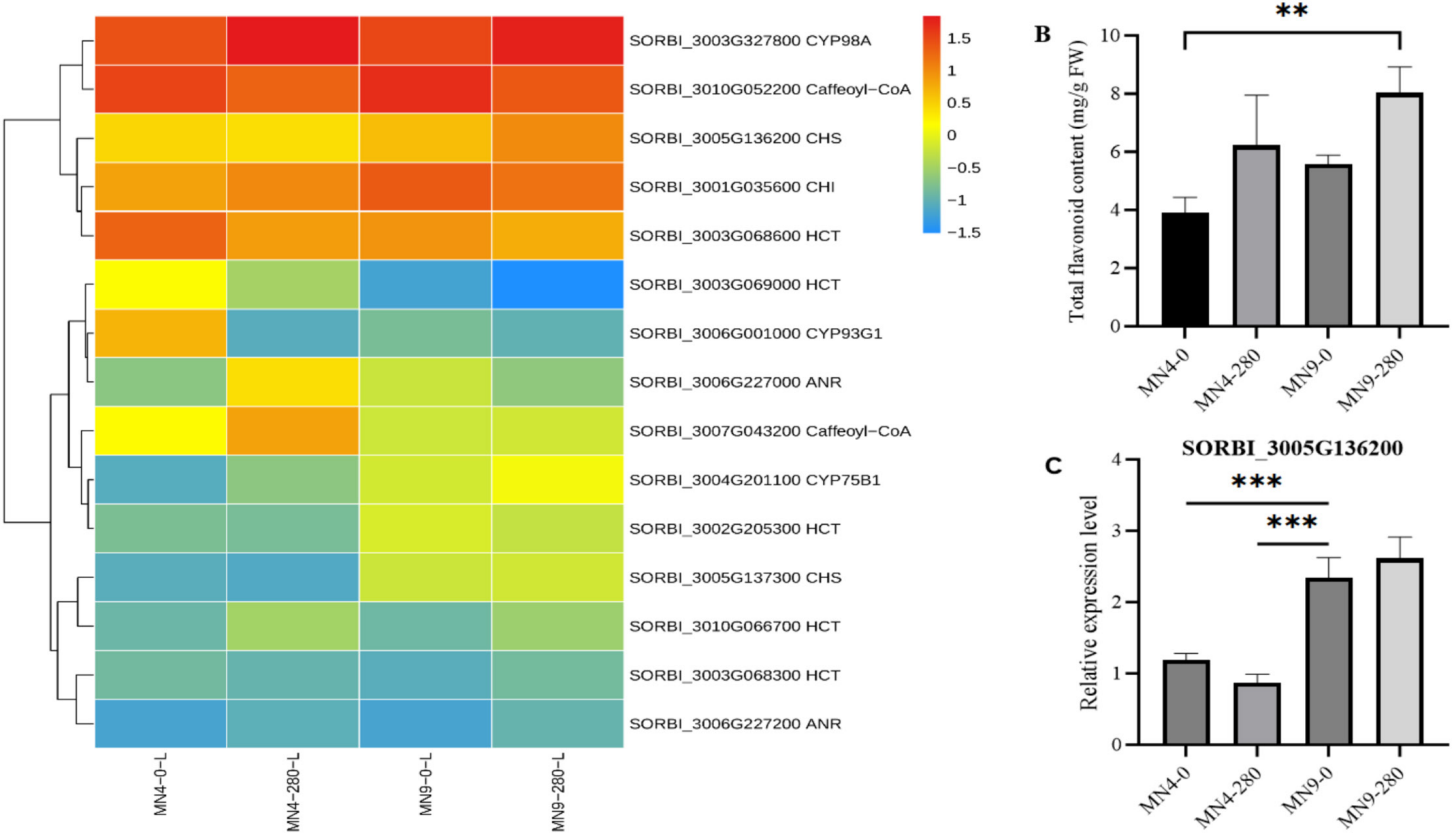
Heatmap of expression levels of key genes in the flavonoid biosynthesis pathway across treatments. (A) Heatmap of key genes in the flavonoid biosynthesis pathway. Colors represent row‐wise Z‐score normalized log_2_(FPKM) values; red indicates high expression, and blue indicates low expression. (B) Relative expression levels of selected genes validated by qPCR under control and salt stress treatments. (C) Flavonoid content under control and salt stress treatments. *Note:* ** indicates significance at *p* < 0.01.

To elucidate the molecular and physiological mechanisms underlying differential salt tolerance between the two sorghum‐sudangrass hybrids (MN9 and MN4), KEGG pathway enrichment analysis was performed on leaf transcriptome data obtained under salt stress conditions. The results revealed that MN9 maintained robust basal expression and a high inducible response at upstream and core nodes of key pathways. Chalcone synthase (CHS) is the first committed rate‐limiting enzyme in flavonoid biosynthesis. The CHS‐encoding gene SORBI_3005G136200, along with the downstream cytochrome P450 genes SORBI_3003G327800 (*CYP98A*) and SORBI_3004G201100 (*CYP75B1*), exhibited higher expression levels in MN9 compared with MN4 under salt stress (Figure 6A). To validate these findings, SORBI_3005G136200 was selected for qRT‐PCR analysis; the expression of this gene was significantly higher in MN9 than in MN4 under salt stress conditions (Figure 6C). Additionally, the flavonoid content was quantified in both cultivars. Flavonoid levels increased in both varieties following salt stress, with MN9 accumulating the most flavonoids among all treatment groups.

### 3.7 The Cyanoamino Acid Metabolic Pathway Exhibits Synergistic and Efficient Cyanide Detoxification Potential in the Salt‐Tolerant Cultivar MN9

**FIGURE 7:**
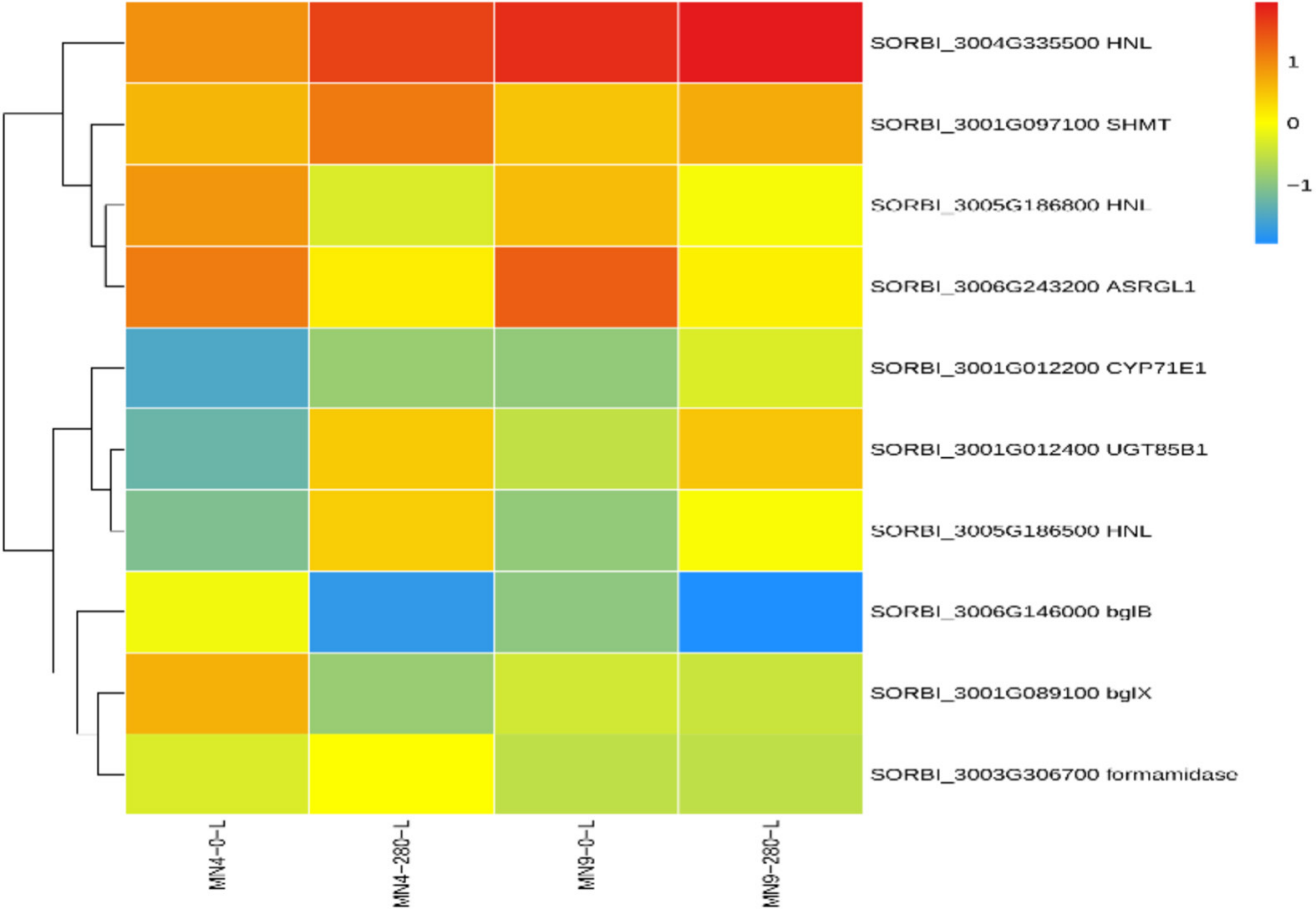
Differential expression of key genes in the cyanoamino acid metabolism and flavonoid biosynthesis pathways under salt stress. Colors denote row‐wise Z‐score normalized log_2_(FPKM) values; red indicates high expression, and blue indicates low expression.

Ten genes associated with the cyanoamino acid metabolic pathway, including *HNL*, *UGT85B1*, and *SHMT*, were implicated in the salt stress tolerance response of sorghum‐sudangrass hybrids (Table 4). Hydroxynitrile lyase (*HNL*) is the initial and rate‐limiting enzyme in the plant cyanide detoxification pathway. The core gene SORBI_3004G335500, directly involved in cyanide detoxification, exhibited significantly higher expression in MN9 compared with MN4 under salt stress, and other genes throughout the cyanide detoxification cascade also showed coordinated upregulation in MN9. The gene SORBI_3001G012400, encoding uridine diphosphate glucosyltransferase (UGT85B1), and the hub gene SORBI_3001G097100 were both strongly upregulated in both cultivars under salt stress. The latter encodes serine hydroxymethyltransferase (SHMT), which not only provides substrates for this pathway but also bridges cyanide detoxification and antioxidant defense systems by generating glycine, a precursor for glutathione synthesis (Figure 7). This gene may represent a core regulatory node through which MN9 achieves stress resistance.

**TABLE 4:** Expression levels of key genes involved in the cyanoamino acid metabolism pathway of sorghum‐sudangrass hybrids under different salt treatments.

Gene ID	MN4‐0‐L	MN4‐280‐L	MN9‐0‐L	MN9‐280‐L
SORBI_3001G012200 CYP71E1	3.43	14.90	14.48	40.63
SORBI_3001G012400 UGT85B1	4.99	80.56	23.45	125.24
SORBI_3001G089100 bglX	68.19	14.54	27.30	32.31
SORBI_3001G097100 SHMT	65.83	184.71	77.61	172.09
SORBI_3003G306700 formamidase	19.35	46.15	22.83	28.40
SORBI_3004G335500 HNL	98.28	334.35	338.12	997.82
SORBI_3005G186500 HNL	6.53	78.03	14.37	58.85
SORBI_3005G186800 HNL	93.18	30.21	83.76	58.14
SORBI_3006G146000 bglB	25.79	4.19	13.85	3.06
SORBI_3006G243200 ASRGL1	122.74	53.69	207.26	73.73

### 3.8 qRT‐PCR Validation

**FIGURE 8:**
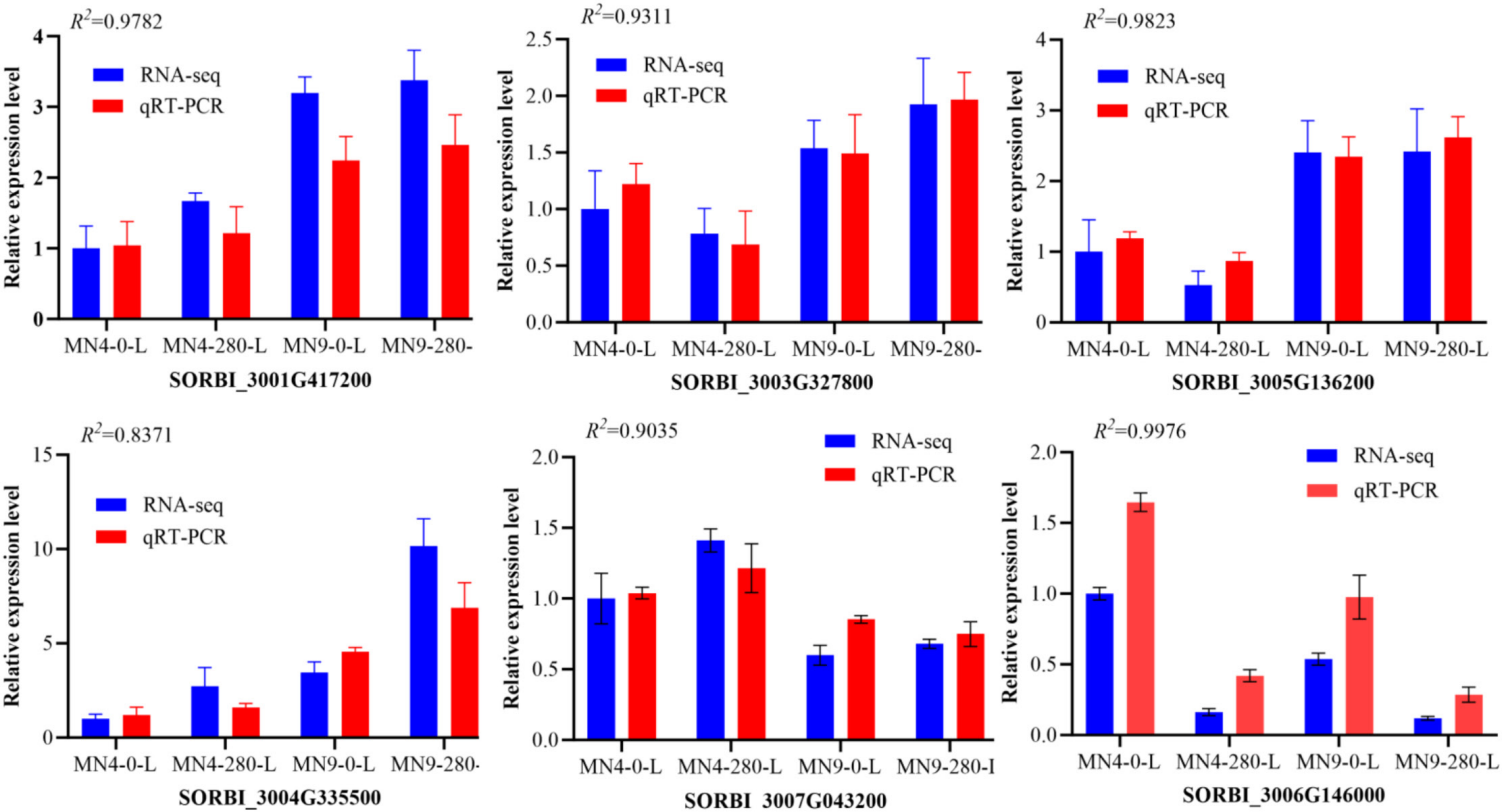
Quantitative real‐time PCR validation of selected salt‐responsive genes in sorghum‐sudangrass hybrid leaves. *Note:* Each subgraph shows the relative expression levels determined by RNA‐seq (blue bars) and qRT‐PCR (red bars) under control (0‐mmol/L NaCl) and salt stress (280‐mmol/L NaCl) conditions. MN4 and MN9 represent the two sorghum‐sudangrass hybrid lines; ‐0‐L and ‐280‐L denote the control and salt‐stressed leaf samples, respectively. The coefficient of determination (*R* ^2^) indicates the correlation between the two methods. Data are presented as mean ± standard error (*n* = 3).

To verify the reliability of the transcriptome sequencing results, four genes associated with photosynthesis, flavonoid biosynthesis, and cyanoamino acid metabolism pathways were randomly selected for qRT‐PCR validation (Figure 8). Correlation analysis between qRT‐PCR and RNA‐Seq data revealed significant positive correlations for all four genes (*p* < 0.05), confirming the accuracy and reliability of the transcriptome sequencing results.

## 4 Discussion

### 4.1 MN9 Exhibits Higher Salt Tolerance Than MN4

Sorghum‐sudangrass hybrids inherit strong salt tolerance from their parental sorghum (Vasilakoglou et al. 2011), and different genotypes exhibit considerable variation in physiological responses under salt stress (Nawaz et al. 2010; Sui et al. 2015). In the present study, six salt concentration gradients were established to evaluate POD activity, proline content, and SPAD values in two cultivars, MN9 and MN4. The results demonstrated that 280‐mM NaCl represents the critical threshold for distinguishing salt tolerance characteristics between the two cultivars. Furthermore, the salt‐tolerant genotype MN9 and salt‐sensitive genotype MN4 displayed significant differences in both phenotypic and physiological responses under salt stress, consistent with the salt tolerance differentiation patterns observed among sweet sorghum genotypes. The significant accumulation of proline in MN9 represents one mechanism for coping with osmotic stress under salinity. Previous studies have demonstrated that the proline concentration increases as salt stress intensifies. Proline can not only quench reactive oxygen species (ROS) but also maintain the stability of antioxidant enzymes and the antioxidant defense system in plants (Hoque et al. 2008). In olive, proline has been shown to improve salt tolerance by enhancing antioxidant enzyme activities, photosynthetic capacity, and overall plant growth (Ben Ahmed et al. 2010). Under salt stress, excessive ROS accumulation triggers membrane lipid peroxidation, impairing cell membrane integrity and disrupting cellular physiological homeostasis. Upon salt exposure, the salt‐tolerant genotype MN9 exhibited distinctly elevated POD activity, conferring superior enzymatic antioxidant capability to sustain cellular stability. It has been documented that cucumber could rapidly activate enzymatic antioxidant systems including POD under salinity stress, which synergistically eliminates O_2_^−^ and H_2_O_2_ and mitigates ROS‐induced oxidative injury (Sarwar et al. 2021). Physiological evaluation revealed obvious genotypic differences in antioxidant responses and salt stress adaptation between the two materials.

### 4.2 The Flavonoid Biosynthesis Pathway Contributes to the Enhancement of Salt Tolerance in Sorghum‐Sudangrass Hybrid Leaves

Flavonoids are a class of polyphenolic secondary metabolites that protect plants against both biotic and abiotic stresses (Ahad et al. 2018). KEGG enrichment analysis in the present study revealed that DEGs were predominantly associated with flavonoid biosynthesis and secondary metabolite pathways. Salt stress can induce differential regulation of plant secondary metabolites (Gan et al. 2021; Song et al. 2023), thereby participating in the salt tolerance response process in sorghum‐sudangrass hybrids. This metabolic response characteristic has been widely reported in crops such as soybean (Li 2020). The flavonoid metabolic pathway is particularly important for the resistance of plants to abiotic stresses (Lea et al. 2007; Wu et al. 2026). CHS is a key rate‐limiting enzyme in the flavonoid biosynthesis pathway and acts at the initial regulatory site of the whole pathway. In this study, we identified a pivotal gene *SORBI_3005G136200* (CHS), which was confirmed through sequence annotation and cross‐species BLAST alignment as a member of the sorghum *CHS* gene family, subsequently designated as *SbCHS5*. Under salt stress, this gene exhibited higher expression levels in the salt‐tolerant genotype MN9. Moreover, the relative expression trend of the *CHS* gene was consistent with the change in flavonoid content, confirming that the differential expression of *SbCHS5* acts as a key regulatory factor underlying the difference in flavonoid accumulation between the two genotypes. Further genomic analysis revealed the existence of multiple *CHS* homologous genes in sorghum, forming a multigene family, with *SbCHS5* identified as the major functional copy involved in the salt stress response of sorghum‐sudangrass hybrids. Protein sequence alignment showed that the protein encoded by *SbCHS5* shares up to 99% sequence identity with other *CHS* family members in sorghum, and 94% identity with *CHS* homologous proteins from gramineous model crops such as maize and rice. Preserves fully intact catalytic domains across species without deletions. Previous studies have reported that CHS and CHI genes serve as entry points to the flavonoid biosynthesis pathway, and that *CHS* and *CHI* expression in citrus promotes flavonoid accumulation (Wang et al. 2010; Zhang et al. 2024). Flavonoid accumulation under salt stress has been identified as an important mechanism for the adaptation of rice roots to saline conditions (Chandran et al. 2019). Similarly, KEGG analysis of the salt‐tolerant halophyte *Reaumuria trigyna* demonstrated enhanced phenylpropanoid and flavonoid metabolism under salt stress, which represent key salt tolerance mechanisms in this species (Dang 2013).

### 4.3 Cyanogenic Amino Acid Metabolism Pathway Synergistically Regulates Salt Tolerance Defense Capacity in Sorghum‐Sudangrass Hybrids

**FIGURE 9:**
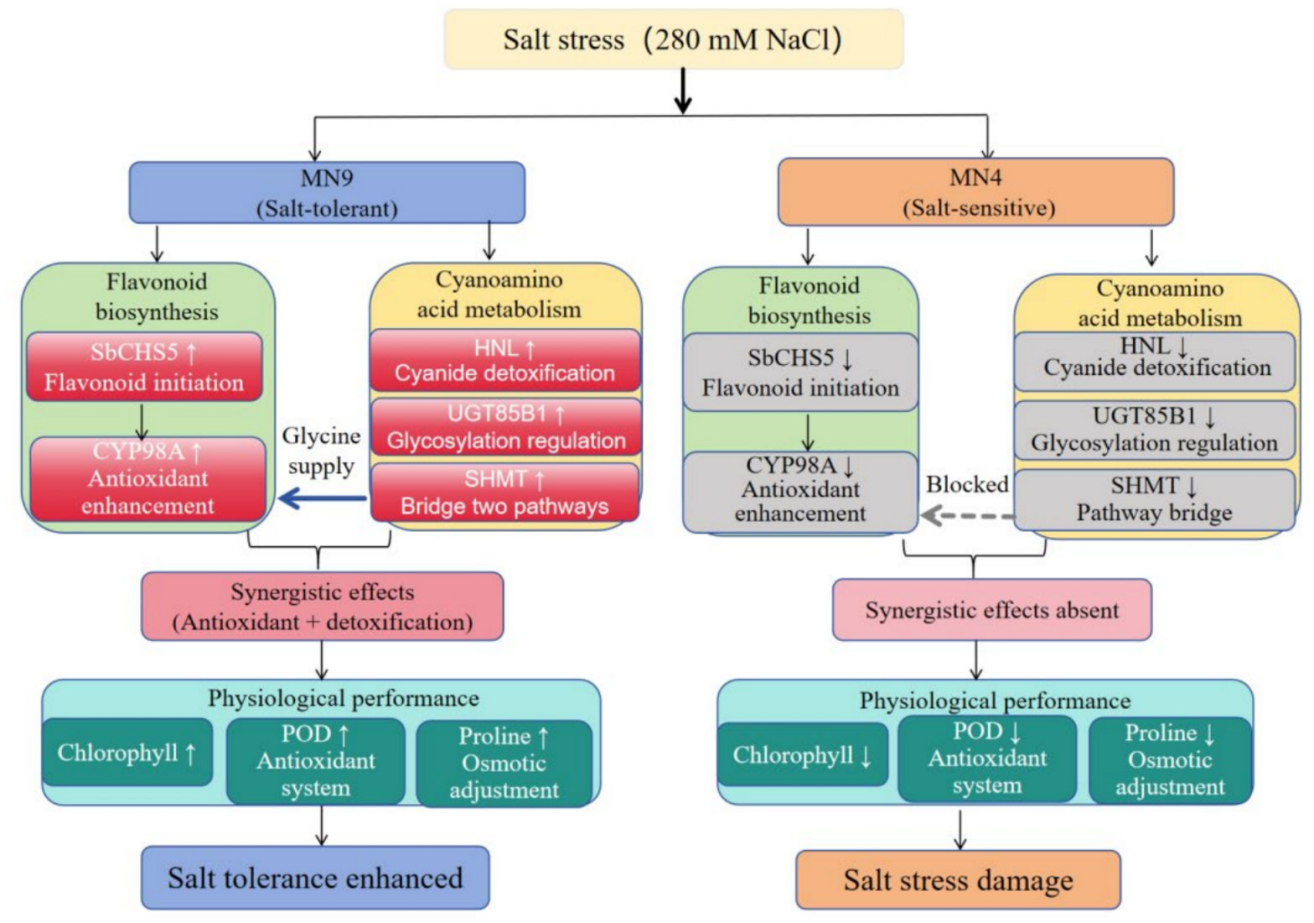
Coordinated regulatory mechanisms in response to salt stress between salt‐tolerant and salt‐sensitive varieties of sorghum‐sudangrass hybrid. *Note:* Under salt stress, key genes in flavonoid biosynthesis and cyanoamino acid metabolism were coordinately upregulated in MN9, with *SHMT* mediating pathway crosstalk to enable “detoxification‐antioxidant” synergism. This maintained physiological homeostasis and enhanced salt tolerance. In MN4, gene expression was inhibited, crosstalk was blocked, and synergistic effects were absent, resulting in severe salt stress damage.

Cyanoamino acids are nonprotein amino acids containing a cyano group (–CN) in their molecular structure, with β‐cyanoalanine (β‐CA) as the core representative. These compounds are widely distributed in plants and are primarily involved in critical physiological processes, including cyanide detoxification, nitrogen metabolism, and stress responses (Ansari et al. 2019). Cyanide may function as a signaling molecule regulating certain metabolic processes (Bogatek et al. 1991; Grossmann 1996; Siegień and Bogatek 2006). Numerous studies have demonstrated that cyanide serves as a mediator regulating seed germination and dormancy release in *Oryza sativa* , *Malus domestica* , *Helianthus tuberosus* , and *A. thaliana* (Bogatek et al. 1991; Cohn and Hughes 1986; Fol et al. 1989; Bethke et al. 2006) and enhances salt stress tolerance in tobacco (Yu et al. 2021; Yu et al. 2020). However, the role of this pathway in salt tolerance regulation in sorghum‐sudangrass hybrids has not yet been systematically elucidated. This study reveals that the cyanoamino acid metabolic pathway serves as a key regulatory module in sorghum‐sudangrass hybrid's response to salt stress. Its coordinated regulation with the flavonoid biosynthesis pathway represents one of the core mechanisms underlying salt tolerance in the resistant cultivar MN9. Under salt stress, the β‐hydroxy nitrile lyase‐encoding gene *SORBI_3004G335500* (*HNL*) was significantly upregulated in MN9. The enzyme encoded by this gene acts as the initial key enzyme in the cyanide detoxification pathway, efficiently hydrolyzing cyanogenic glycosides produced during stress‐induced ethylene synthesis. This process effectively reduces the accumulation of toxic intermediates such as hydrogen cyanide (HCN), thereby preventing cellular respiration inhibition and metabolic disruption. Concurrently, the glycosyltransferase‐encoding gene *UGT85B1* and serine hydroxymethyltransferase‐encoding gene *SHMT* exhibited coordinated upregulation patterns in MN9. *UGT85B1* participates in the glycosylation modification of cyano compounds to reduce their biological toxicity. Meanwhile, *SHMT* functions as a critical interpathway hub that provides glycine as precursor material for glutathione synthesis. This mechanism directly bridges the cyanide detoxification and antioxidant systems, establishing a coordinated “detoxification‐antioxidant” regulatory network. This node likely represents the core component of MN9's multilayered stress defense system (Figure 9). In contrast, the key genes involved in cyanoamino acid metabolism including *HNL*, *UGT85B1*, and *SHMT* were markedly suppressed in salt‐sensitive cultivar MN4. The interpathway connection was blocked, failing to form effective synergy with the flavonoid pathway. This resulted in insufficient detoxification and antioxidant capacity, and ultimately aggravated salt‐induced damage. Studies on salt tolerance of banana seedlings also demonstrated that cyanoamino acid metabolism is one of the crucial pathways in melatonin‐mediated salt stress responses (Wei et al. 2022). This study further demonstrates that in sorghum‐sudangrass hybrids, this pathway can achieve multilayered stress protection via hub gene‐mediated cross‐pathway coordination, providing new perspectives for understanding salt tolerance mechanisms in these crops.

## 5 Conclusions

Following salt stress, the salt‐tolerant variety MN9 maintained higher chlorophyll content, enhanced proline accumulation, and increased POD activity, demonstrating significant physiological advantages over the salt‐sensitive cultivar MN4. Comparative transcriptome analysis between salt‐tolerant and salt‐sensitive varieties revealed that MN9 employs a more refined gene regulatory network to cope with salt stress. Key photosynthetic genes (*petF*) exhibited stable expression, whereas pivotal flavonoid biosynthesis genes (*CHS* and *CYP98A*) were specifically activated, accompanied by flavonoid accumulation, which enhances antioxidant capacity. Additionally, key genes in the cyanoamino acid metabolism pathway, *HNL* (SORBI_3004G335500), *UGT85B1*, and *SHMT*, showed coordinated upregulation, improving cyanide detoxification and nitrogen recycling efficiency. Notably, *HNL* is the first validated gene demonstrated to play a critical role in sorghum‐sudangrass salt tolerance. This study provides an important theoretical foundation for mining salt tolerance genes and breeding stress‐resilient varieties.

## Author Contributions

Y.S. conceived and designed the study. Y.S., Z.L., S.L., and W.C. performed the experiments. Y.S. wrote the manuscript. Z.L. and J.M. assisted with experimental procedures. Y.S. is the corresponding author, as designated by the institute, and provided funding support for this study. All authors have read and approved the final version of the manuscript.

## Funding

This work was supported by grants from the Special Fund for the “Grassland Talents” Project of Inner Mongolia Autonomous Region (CYYC202402) and the Doctoral Innovation Research Fund of Jining Normal University (jsbsjj2304).

## Conflicts of Interest

The authors declare no conflicts of interest.

## Data Availability

The data that support the findings of this study are available on request from the corresponding author. The data are not publicly available due to privacy or ethical restrictions.
